# Tea Consumption and Incidence of Type 2 Diabetes in Europe: The EPIC-InterAct Case-Cohort Study

**DOI:** 10.1371/journal.pone.0036910

**Published:** 2012-05-30

**Authors:** The InterAct Consortium

**Affiliations:** German Diabetes Center -, Leibniz Center for Diabetes Research at Heinrich Heine University Duesseldorf, Germany

## Abstract

**Background:**

In previous meta-analyses, tea consumption has been associated with lower incidence of type 2 diabetes. It is unclear, however, if tea is associated inversely over the entire range of intake. Therefore, we investigated the association between tea consumption and incidence of type 2 diabetes in a European population.

**Methodology/Principal Findings:**

The EPIC-InterAct case-cohort study was conducted in 26 centers in 8 European countries and consists of a total of 12,403 incident type 2 diabetes cases and a stratified subcohort of 16,835 individuals from a total cohort of 340,234 participants with 3.99 million person-years of follow-up. Country-specific Hazard Ratios (HR) for incidence of type 2 diabetes were obtained after adjustment for lifestyle and dietary factors using a Cox regression adapted for a case-cohort design. Subsequently, country-specific HR were combined using a random effects meta-analysis. Tea consumption was studied as categorical variable (0, >0-<1, 1-<4, ≥4 cups/day). The dose-response of the association was further explored by restricted cubic spline regression. Country specific medians of tea consumption ranged from 0 cups/day in Spain to 4 cups/day in United Kingdom. Tea consumption was associated inversely with incidence of type 2 diabetes; the HR was 0.84 [95%CI 0.71, 1.00] when participants who drank ≥4 cups of tea per day were compared with non-drinkers (*p*_linear trend_ = 0.04). Incidence of type 2 diabetes already tended to be lower with tea consumption of 1-<4 cups/day (HR = 0.93 [95%CI 0.81, 1.05]). Spline regression did not suggest a non-linear association (*p_non-linearity_* = 0.20).

**Conclusions/Significance:**

A linear inverse association was observed between tea consumption and incidence of type 2 diabetes. People who drink at least 4 cups of tea per day may have a 16% lower risk of developing type 2 diabetes than non-tea drinkers.

## Introduction

Increasing our understanding of modifiable lifestyle factors associated with the development of type 2 diabetes is important, as the prevalence of diabetes is increasing rapidly [1]. Obesity is a major risk factor for the development of type 2 diabetes [2], but dietary factors may also play a role. One dietary factor of interest is tea consumption. Tea consumption may lower the risk of type 2 diabetes by influencing glucose digestion, glucose uptake, and by protecting beta-cells from free-radical damage [3]–[5]. This beneficial effect may be due to the polyphenols present in tea.

A meta-analysis, including nine cohort studies, reported that drinking at least 4 cups of tea per day was associated with a 20% lower risk, whereas drinking >0-<1 or 1–3 cups per day did not lower the risk of diabetes compared with non-tea drinkers [6]. In line with this, no association was observed when tea consumption was studied as continuous variable. This may indicate that the protective effect of tea is restricted to people with a high tea consumption, although a potential biological mechanism has not yet been described.

Studies in which tea consumption is low, therefore, may not observe an association between tea consumption and risk of type 2 diabetes. This is supported by three out of the four additional cohort studies, which were published after the meta-analysis [7]–[10]. Two of those studies, in which the highest tea consumption category was relatively low, at least 3 cups per day [7] or at least 2 cups per day [8], did not observe an association between tea consumption and risk of diabetes, whereas one study, in which the highest tea consumption category was more than 5 cups per day, observed a substantial lower risk [9]. In contrast, one study did not observe an association, even though the highest category of tea consumption included participants who drank at least 4 cups per day [10]. So, to date it is unclear whether or how tea consumption is associated with risk of type 2 diabetes.

Therefore, we investigated the association between tea consumption and incidence of type 2 diabetes in European citizens who were part of the EPIC-InterAct project. The size of the study is comparable with the meta-analyses reported to date on this topic [6], [11] and provides the opportunity to explore a potential non-linear association between tea consumption and risk of type 2 diabetes across European countries.

## Methods

### Ethics statement

The study complied with the Declaration of Helsinki. The Internal Review Board of the International Agency for Research on Cancer and the Institutional Review Board of all centers, i.e., France, Heidelberg, Postdam, Copenhagen, Aarhus, Asturias, Granada, San Sebastian, Murcia, Navarro, Cambridge, Oxford, Imperial, Florence, Milan, Ragusa, Turin, Naples, Bilthoven, Utrecht, Malmö, and Umeä, approved the EPIC study. Written consent was obtained from each EPIC participant at enrolment into the study.

### Population

The EPIC-InterAct project is a case-cohort study embedded in the EPIC study. The EPIC study is a prospective study conducted in 10 European countries [12]. Eight countries also participated in the EPIC-InterAct project (Spain, Italy, Sweden, France, Denmark, Germany, The Netherlands, and United Kingdom), with a total of 26 centers. The rationale and design of the EPIC-InterAct project has been described in detail elsewhere [13]. In short, a center-stratified random sample of 16,835 participants, aged 20–79 years, was taken as sub-cohort. Subsequently, a number of 548 participants with prevalent diabetes and 133 with unknown diabetes status were excluded, resulting in 16,154 participants. After verification of all eligible EPIC participants for diabetes incidence, 12,403 verified cases were obtained of which 778 belongs to the sub-cohort.

Our analysis included 26,039 participants of the 27,779 participants included in the EPIC-InterAct-project, because we excluded in consecutive order 117 participants without dietary data, 619 with unreliable food intake data (top and bottom 1% of the distribution of energy intake to basal energy requirement assessed by WHO/FAO/UNU equation including weight and height) [14], and 955 with missing information about potential confounders which were included in the final model (289 without physical activity measurements, 134 without information on smoking status, 367 without information on educational status, and 165 without information on body mass index (BMI)). Furthermore, since few participants from Spain (*n* = 39) and Italy (*n* = 10) drank ≥4 cups of tea per day, country-specific hazard ratios (HR) comparing ≥4 vs. 0 cups per day could not be obtained in Spain and Italy. Therefore, those 49 participants were also excluded.

### Dietary intake including tea consumption

Dietary intake over the last 12 months was assessed by country-specific or center-specific semi-quantitative or quantitative dietary questionnaires, validated within each country [12]. More information about the questionnaires can be found elsewhere [12]. All questionnaires included at least one question about tea consumption. Each center converted the information about tea consumption into grams per day. For the analysis, tea consumption in grams per day was divided by 125 to be able to calculate HR by cups per day. In line with the meta-analysis by Jing *et al.* [6], the frequency of tea consumption was divided into 4 categories: 0, >0-<1, 1-<4, ≥4 cups per day.

### Diabetes incidence

A pragmatic, high sensitivity approach for case ascertainment was used in order to identify all potential incident type 2 diabetes cases and excluding all individuals with prevalent diabetes [13]. Briefly, ascertainment of incident diabetes involved a review of the existing EPIC datasets at each center using multiple sources of evidence including self-report, hospital admissions, linkage to primary care registers, linkage to secondary care registers, linkage to drug registers, and mortality data. Cases in Denmark and Sweden were not ascertained by self-report, but via diabetes and pharmaceutical registers. Hence, all ascertained cases were considered to be verified. To increase the ability to exclude false negatives for countries other than those from Denmark and Sweden, we sought further evidence for all cases with information on incident type 2 diabetes from less than 2 independent sources which have been described in detail elsewhere [13]. Follow-up was censored at the date of diagnosis, the 31st of December 2007, or the date of death, whichever occurred first.

### Non-dietary covariates

Socio-demographic and lifestyle information, e.g., age, sex, education level, smoking status, and physical activity during work and leisure time [15], was obtained with questionnaires at baseline.

Questionnaires were also used to obtain information about diseases of the participant and his family, i.e., history of angina pectoris (not in The Netherlands, Sweden, and one center in Germany), history of myocardial infarction, history of stroke (not in one center in Sweden), presence and/or treatment for hypertension, presence and/or treatment of hyperlipidemia (not in one center in Sweden), and family history of type 2 diabetes (not in Spain, Italy, one center in Germany, and one center in the United Kingdom).

Information about height and weight was obtained using a standard protocol during a visit at the research center at baseline for all participants, except in France and in some of the participants from one center in the United Kingdom. Self-reported or corrected height and weight were used in those centers without measured height and weight [12].

### Statistical analysis

The association between tea consumption (0, >0-<1, 1-<4, ≥4 cups/day) and risk of type 2 diabetes was examined by country using modified Cox proportional hazard models with age as underlying time scale. The models were modified for the case-cohort design according to the Prentice method [16]. In order to adjust for time to follow-up, age at recruitment (1-year categories) was included as stratum variable. Summary HR and 95% confidence intervals (95%CI) were obtained by pooling country-specific HR using random effects meta-analyses and visualized in forest plots. Between country heterogeneity was assessed by *I* ^2^ statistic, i.e., the percentage of variation in the HR attributable to between country heterogeneity [17].

To obtain adjusted country-specific HR, four Cox models were constructed. Variables included in these models were considered main potential confounders in the association between tea consumption and risk of type 2 diabetes based upon literature. Model 1 included, in addition to tea consumption, four other covariates: sex, smoking status (never, former, current), physical activity level (inactive, moderately inactive, moderately active, and active), and education level (lowest, secondary, and highest). Model 2 was similar to model 1 with additional adjustments for energy and intake of seven nutrients: protein (energy-%), carbohydrates (energy-%), saturated fatty acids (energy-%), mono-unsaturated fatty acids (energy-%), poly-unsaturated fatty acids (energy-%), alcohol (0, >0–6, >6–12, >12–24, and >24 g/d), and fiber (g/d). Model 3 was similar to model 2 with additional adjustments for intake of drinks: coffee (g/d), juices (g/d), soft-drinks (g/d), and milk (g/d). Model 4 was similar to model 3 with additional adjustment for BMI (kg/m^2^).

After investigating the association between tea consumption as a categorical variable and risk of type 2 diabetes, the dose-response of the association was further explored by studying linear trends across categories, by restricted cubic spline regression, and by studying consumption of tea (cups per day) as a continuous variable. To test for linear trends across categories, the median value of each category of tea consumption was modeled as a continuous variable. The restricted cubic spline regression was performed using SAS Macro RCS, which was also based on the modified Cox proportional hazard regression. The knots were located at 1, 4, 7 cups per day and non-tea drinkers were used as reference group. This analysis was adjusted as described for model 4.

Potential effect modification was investigated by including in the models an interaction term between tea categories and sex or tea categories and BMI categories and by studying the association between tea consumption and risk of type 2 diabetes by sex (men: *n* = 11,249; women: *n* = 15,030) and by BMI (Normal: BMI<25.0 kg/m^2^: *n* = 8,267; Overweight: BMI≥25.0-<30.0 kg/m^2^: *n* = 10,840; Obese: BMI≥30.0 kg/m^2^: *n* = 6,932).

To investigate the robustness of the associations, sensitivity analyses were performed by excluding one by one participants for each of the following diseases at baseline: a history of stroke (*n* = 261), a history of angina pectoris (*n* = 521), a history of a myocardial infarction (*n* = 545), hypertension (*n* = 5,682), and hyperlipidemia (*n* = 4,362). Furthermore, participants with a family history of diabetes (*n* = 2,928) and who developed type 2 diabetes within 2 years (*n* = 955) were excluded in the sensitivity analyses. We excluded these participants in the sensitivity analyses, because they may have changed recently their diet. In centers which did not obtain information about disease history, participants were considered as not having the disease at baseline.

Analyses were carried out using the statistical software program SAS version 9.1, except for the random effects meta-analyses which were conducted in STATA 11.0. A two-sided p-value≤0.05 was considered as statistically significant for all analyses.

## Results

**Figure 1:**
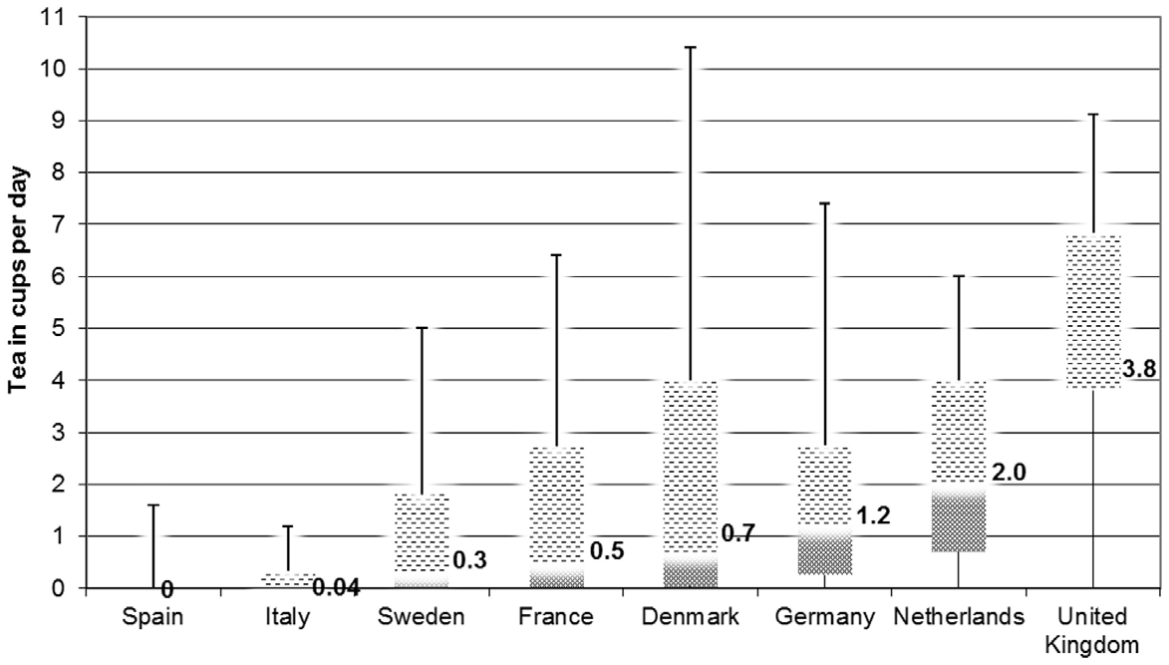
Tea consumption based on data from a food frequency questionnaire in the sub-cohort of the EPIC-InterAct project by country (*n* = 15,227). Bar represents median (p25–p75); error line represents p5 till p95.

Overall, 64% of this European study population reported that they drank tea. The highest median total tea consumption was observed in the United Kingdom (3.8 (IQR 3.8–6.8) cups per day); the lowest in Spain (0.0 (IQR 0-0) cups per day) (**Figure 1**). The median total tea consumption among drinkers was 1.2 (IQR 0.3–3.7) cups per day. In general, participants with higher tea consumption had a lower BMI, had a higher level of education, and smoked less (**Table 1**). Intake of carbohydrate and saturated fatty acids was higher, whereas intake of mono-saturated fatty acids was lower across tea categories. Tea drinkers drank less alcohol, but more coffee, soft drinks, and juices than non-tea drinkers.

**Table 1:** Characteristics of the sub-cohort of the EPIC-InterAct project by categories of tea consumption (*n* = 15,227).

	None	>0-<1	1-<4	≥4
		(cups/day)	(cups/day)	(cups/day)
	(n = 5,458)	(n = 4,032)	(n = 3,444)	(n = 2,293)
Age (years)	51.7 (8.6)	51.4 (9.1)	53.0 (9.7)	54.7 (8.8)
Body mass index (kg/m^2^)	27.0 (4.3)	25.8 (4.0)	25.5 (4.0)	25.0 (3.8)
Male (%)	40.5	41.3	32.7	33.6
Country (%)				
*Spain*	83.0	7.5	9.5	0
*Italy*	43.1	44.2	12.8	0
*Sweden*	33.6	30.5	28.2	7.7
*France*	36.7	21.1	27.4	15.1
*Denmark*	17.3	38.8	9.8	34.1
*Germany*	6.1	39.6	36.5	17.8
*Netherlands*	8.3	18.7	42.1	31.0
*United Kingdom*	5.2	8.9	39.0	46.9
Smoking (% current)	31.9	27.5	19.6	18.8
Education level (% high)	13.7	21.7	25.3	28.8
Physical activity (% inactive)	31.6	20.1	20.4	15.6
Hypertension (%)	17.5	19.1	20.3	17.4
Hyperlipidaemia (%)^1^	17.9	15.8	17.0	10.3
Family history of diabetes (%)^2^	13.9	17.1	16.9	17.9
Stroke (%)^3^	0.7	0.9	1.1	0.7
Angina Pectoris (%)^4^	1.3	2.4	3.1	2.4
Myocardial infarct (%)	1.1	1.4	1.6	1.7
**Dietary intake**				
Total energy (kcal/d)	2188 (662)	2140 (645)	2067 (591)	2124 (603)
Protein (energy%)	18.0 (3.2)	16.4 (2.8)	16.5 (3.1)	16.6 (2.9)
Fat (energy%)	35.4 (6.1)	34.6 (5.7)	34.6 (5.8)	34.0 (5.6)
Saturated fatty acids	12.4 (3.6)	13.6 (3.3)	13.7 (3.3)	13.8 (3.1)
Mono-unsaturated fatty acids	14.6 (3.7)	13.0 (3.1)	12.3 (3.0)	11.3 (2.2)
Poly-unsaturated fatty acids	5.6 (2.1)	5.2 (1.6)	5.8 (1.8)	5.9 (1.8)
Carbohydrate (energy%)	42.2 (7.2)	44.7 (6.9)	45.1 (6.8)	45.5 (6.7)
Fiber (g/d)	22.7 (8.1)	22.1 (7.4)	22.4 (7.2)	24.6 (8.0)
Alcohol (%>24 g/d)	22.6	19.9	15.1	15.5
Coffee (g/d)	154 (60–400)	400 (130–700)	363 (125–525)	375 (86–500)
Soft drinks (g/d)	0 (0–29)	16 (0–86)	14 (0–90)	16 (0–90)
Juices (g/d)	1 (0–25)	22 (3–94)	40 (4–120)	29 (3–100)
Milk (g/d)	180 (61–300)	138 (25–271)	150 (25–295)	193 (36–387)

Values are expressed as Mean (Standard Deviation), Median (p25–p75), or percentage.

1 Based on *n* = 13,345, because information about hyperlipidemia was not collected in one center of Sweden.

2 Based on *n* = 8,802, because information about family history of diabetes was not collected in Italy, Spain, one center of Germany, and one center of the United Kingdom.

3 Based on *n* = 14,262, because information about a history of stroke was not collected in one center of Sweden.

4 Based on *n* = 10,168, because information about a history of angina was not collected in Sweden, The Netherlands, and one center of Germany.

**Figure 2:**
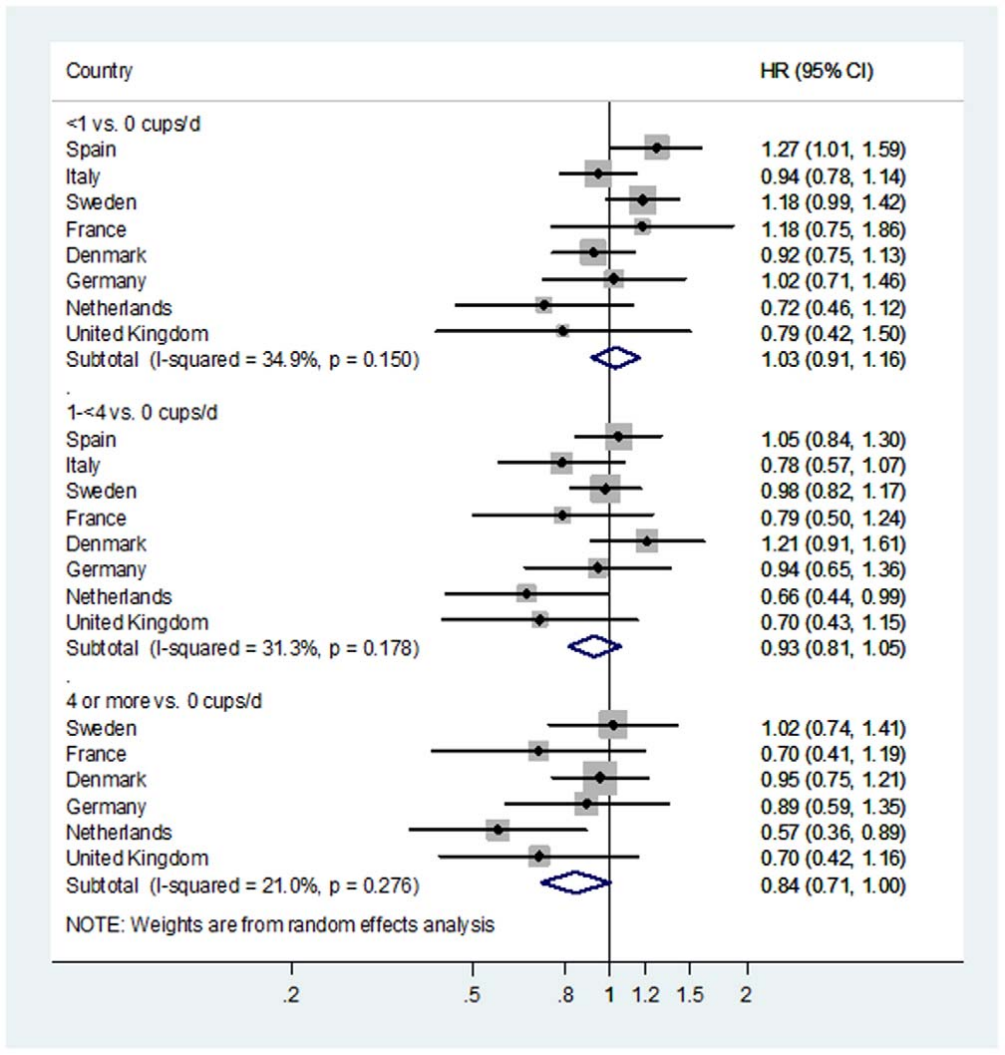
Association between tea consumption as a categorical variable (>0-<1 vs. 0, 1-<4 vs. 0, ≥4 vs. 0 cups/d) based on data from a food frequency questionnaire and risk of type 2 diabetes (*n* = 26,039). Country-specific Hazard Ratios (HR) and 95% Confidence Intervals (95%CI) were pooled using random effects meta-analyses. HR were adjusted for sex, smoking status, physical activity level, education level, intake of energy, protein, carbohydrates, saturated fatty acids, mono-unsaturated fatty acids, poly-unsaturated fatty acids, alcohol, fiber, coffee, juices, soft-drinks, milk, and body mass index.

When tea consumption was divided into categories and country-specific HR were combined, the crude analysis showed that, for all categories, participants who drank tea had a lower risk of developing type 2 diabetes compared with non-tea drinkers (**Table 2**). The risk estimates for type 2 diabetes in all categories of tea consumption were attenuated slightly in model 1. Additional adjustment for the intake of nutrients (model 2) and drinks (model 3) did not affect the risk estimates. Adjustment for BMI (model 4), however, attenuated the risk estimates further, but risk of type 2 diabetes was still 16% lower in participants drinking at least 4 cups of tea per day compared with non-tea drinkers (HR_≥4 cups/d vs. 0_ = 0.84 [95%CI 0.71, 1.00]; *p*_linear trend_ = 0.04). Risk of type 2 diabetes already tended to be lower among participants drinking 1 to 4 cups per day compared with non-tea drinkers (Model 4 HR_1-<4 cups/d vs. 0_ = 0.93 [95%CI 0.81, 1.05]) (**Table 2** **;** **Figure 2**). No evidence of between country heterogeneity was observed in any category of tea consumption (**Figure 2**).

**Table 2:** Hazard Ratios (HR) and 95% confidence intervals (95%CI) for incident type 2 diabetes by categories of tea consumption in cups per day (*n* = 26,039).

				Crude		Model 1		Model 2		Model 3		Model 4	
	N total	Cases	Median	HR	(95% CI)	HR	(95% CI)	HR	(95% CI)	HR	(95% CI)	HR	(95% CI)
0	9,499	4,389	0	1		1		1		1		1	
>0-<1	7,060	3,197	0.23	0.89	(0.80, 0.99)	0.93	(0.81, 1.07)	0.96	(0.84, 1.10)	0.97	(0.85, 1.10)	1.03	(0.91, 1.16)
1-<4	5,751	2,437	2.00	0.77	(0.66, 0.90)	0.83	(0.69, 0.99)	0.85	(0.71, 1.01)	0.84	(0.72, 0.98)	0.93	(0.81, 1.05)
≥4	3,729	1,518	6.84	0.63	(0.50, 0.80)	0.68	(0.52, 0.90)	0.72	(0.53, 0.96)	0.70	(0.54, 0.90)	0.84	(0.71, 1.00)
*p-trend*				*<0.01*		*<0.01*		*<0.01*		*<0.01*		*0.04*	

HR and 95%CI were derived from the modified Cox proportional hazard model by age at baseline and are based on pooled estimates from country specific analyses using a random effects meta-analysis.

Model 1: sex, smoking status, physical activity level, and education level.

Model 2: additional to model 1: intake of energy, protein, carbohydrates, saturated fatty acids, mono-unsaturated fatty acids, poly-unsaturated fatty acids, alcohol, and fiber.

Model 3: additional to model 2: intake of coffee, juices, soft-drinks, and milk.

Model 4: additional to model 3: body mass index.

**Figure 3:**
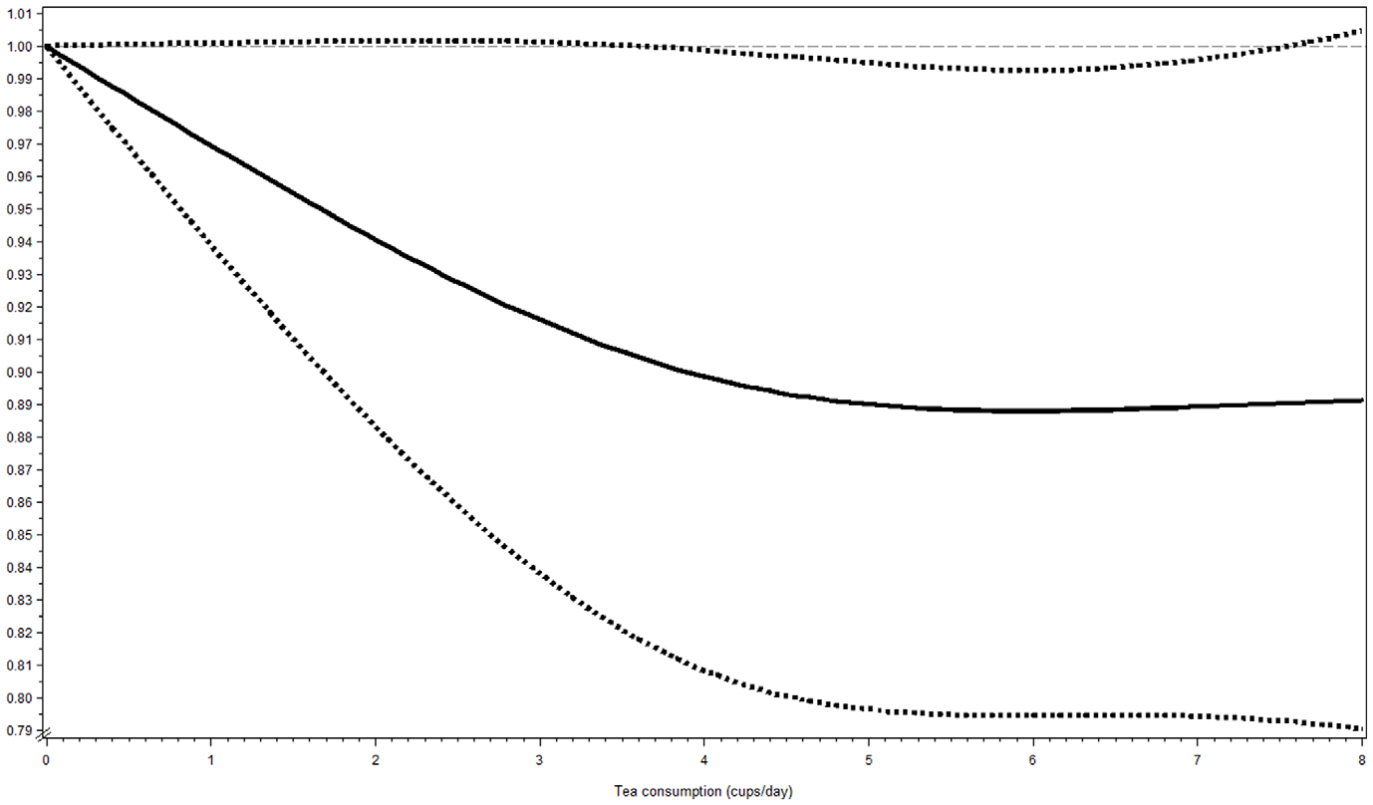
Association between tea consumption based on data from a food frequency questionnaire and risk of type 2 diabetes obtained by spline regression with 3 knots (1, 4, 7 cups per day) and 0 cups per day as reference (*n* = 26,039). Dotted lines represent 95% confidence intervals (95%CI). *P*_non-linearity_ = 0.20. Hazard ratios were adjusted for sex, smoking status, physical activity level, education level, intake of energy, protein, carbohydrates, saturated fatty acids, mono-unsaturated fatty acids, poly-unsaturated fatty acids, alcohol, fiber, coffee, juices, soft-drinks, milk, and body mass index.

The association was further explored by performing spline regression and by studying tea consumption as continuous variable. Cubic spline regression confirmed that risk of type 2 diabetes may be lower with higher intake of tea, although it suggests that the risk may level off after 5 cups per day (*p*_non-linearity_ = 0.20; **Figure 3**). Since the risk reduction may level off, the association between tea consumption on a continuous scale and risk of type 2 diabetes was restricted to participants drinking 5 cups per day or less (*n* = 23,778). This analysis suggested that 1 cup of tea per day was associated with a 3.1% lower risk, which was nearly statistically significant (Model 4: HR 0.97 [95%CI 0.94, 1.00], *p* = 0.06).

Stratified analyses showed that effect modification was not observed for sex (Model 4: *p*_interaction_ = 0.14: *Men*: HR_≥4 cups/d vs. 0_ = 0.89 [95%CI 0.71, 1.10]; *Women*: HR_≥4 cups/d vs. 0_ = 0.82 [95%CI 0.58, 1.16]) and for BMI (Model 4: *p*_interaction_ = 0.26: *BMI<25.0*: HR_≥4 cups/d vs. 0_ = 0.79 [95%CI 0.52, 1.19]; *BMI≥25.0-<30.0*: HR_≥4 cups/d vs. 0_ = 0.87 [95%CI 0.63, 1.20]; *BMI≥30.0*: HR_≥4 cups/d vs. 0_ = 0.79 [95%CI 0.59, 1.05]).

None of the sensitivity analyses changed the results substantially (data not shown).

## Discussion

In this large European population, a linear inverse association was observed between tea consumption and incidence of type 2 diabetes. This significant linear association and the spline regression suggests that a threshold of drinking at least 4 cups of tea per day to lower risk of type 2 diabetes does not appear to exist.

Strengths of our analyses included the ability to study the association between tea consumption and risk of type 2 diabetes in populations from eight European countries, resulting in a larger variation of tea consumption than when these countries were analyzed separately. Our results were also strengthened by the standardized protocol to verify cases of diabetes among countries and to process information about lifestyle and dietary factors. As such, consumption of tea was converted into grams per day for all countries, through which we were able to standardize cup size among countries in the analysis.

Our observation that drinking tea was associated inversely with risk of type 2 diabetes, was in line with the observation of two published meta-analyses equaling the power of our study [6], [11]. We observed a protective association on risk of type 2 diabetes with habitual tea consumption of at least 4 cups per day, Jing *et al.* [6] also with at least 4 cups per day, and Huxley *et al.* [11] with at least 3–4 cups per day. In line with the meta-analysis by Jing *et al.*, we observed that tea drinkers who drank <1 cups per day had a similar risk of type 2 diabetes as non-tea drinkers. Risk of type 2 diabetes, however, already tended to be lower with 1-<4 cups of tea per day in our analysis. Together with the results of the spline regression, our results, therefore, do not support that the protective effect of tea consumption is restricted to participants reporting the highest intake of tea. Therefore, even lower doses may reduce risk of type 2 diabetes.

Our spline regression suggests that the risk reduction leveled off at around 5 cups per day. Potential mechanisms, however, explaining the observed plateau around 5 cups per day are not established. Furthermore, we could not study country-specific associations between a very high tea consumption (>7 cups per day) and risk of type 2 diabetes in a sufficient number of people in our analysis. We already had to exclude Spain and Italy from the highest tea category, because of small numbers in this category. The biological mechanism underlying a beneficial effect of tea, however, is unlikely to differ between Northern and Southern European countries.

The flavonoids present in tea are of importance, because the beneficial effect of tea on risk of type 2 diabetes may be attributable to these components [4], [5]. Catechins, theaflavins, and thearubigins are the most prominent flavonoids in tea. These flavonoids, predominately epigallocatechin gallate (EGCG), have been shown to slow down carbohydrate digestion, to inhibit carbohydrate absorption by competitively binding with the sodium-glucose transporter-1 (SGLT-1), to increase glucose uptake in muscle and fat cells by changes in GLUT-4 expression, to enhance insulin secretion, and to protect beta-cells from free-radical damage [4], [5]. All these pathways can affect glucose concentrations and, thereby, could explain a beneficial effect of tea consumption on risk of type 2 diabetes. Most randomized controlled trials investigating the association between long-term tea consumption and markers of glucose of insulin metabolism, however, showed no associations [18]–[23].

Studies investigating the association between tea consumption and risk of type 2 diabetes may differ by several factors, such as the type of tea consumed, the preparation method used, the cup size used, and the sample size under investigation. The main type of tea consumed may result in discrepancies among studies and among countries in our study, because types of tea differ in chemical composition. Herbal teas may contain less anti-oxidants than black and green tea, because herbal teas, in contrast to black or green tea, are not derived from the *Camellia Sinensis* plant [24]. The difference in anti-oxidant capacity may indicate that the beneficial effect of drinking tea is stronger for black or green tea than for herbal tea. It is likely that tea consumption in our study mainly reflects the intake of black tea, because at the time of dietary data collection in this study, green tea and herbal tea were not as popular as black tea in most countries. Unfortunately, we could not investigate whether the type of tea consumed affects risk of type 2 diabetes differently, because detailed information about type of tea was not collected in most countries.

The preparation method used may also result in discrepancies. The preparation method, including brewing time and substances added, can influence the amount of flavonoids present in a cup of tea [25], [26] and, consequently, the association between tea consumption and risk of type 2 diabetes. Studies, therefore, might find an association at 1-<4 cups of tea per day if the brewing time is long in general, whereas other studies might not find an association if the brewing time is short. Since information about brewing time was lacking in all published studies, including ours, it was not possible to adjust for brewing time. As brewing time was not reported in our study, non-differential misclassification of actual amount of tea consumption could have occurred within countries. Next to a short brewing time, adding milk may also lower the bioavailability of flavonoids due to the interaction between milk proteins and flavonoids present in tea [27]. Five out of six trials, however, showed that adding milk to tea did not affect the bioavailability of tea flavonoids or anti-oxidant capacity after consumption [26], [28]–[32]. Furthermore, the beneficial effect of tea consumption on risk of type 2 diabetes may be counterbalanced by the addition of sugar. Since we did not observe heterogeneity among countries in our study, we do not think that differences in preparation methods among countries have influenced our results.

In our study, tea consumption was associated with a healthier lifestyle, e.g., people who drank tea were more physically active and smoked less. This may indicate that the inverse association between tea consumption and risk of type 2 diabetes reflects a healthier lifestyle rather than tea consumption itself. In our analysis, however, we tried to disentangle the effect of a healthier lifestyle from tea consumption by adjusting the HR for a range of lifestyle and dietary factors, and by excluding people with chronic diseases at baseline in sensitivity analyses. Inclusion of lifestyle and dietary factors apart from BMI, however, did not change the risk estimates much. This may indicate that the effect of a healthier lifestyle could not be adequately adjusted for due to measurement error in these factors. Even though inclusion of BMI into the model attenuated the association between tea consumption and risk of type 2 diabetes, residual confounding by BMI might also be present. As BMI could also be considered as intermediate [33], however, caution should be taken when interpreting the model including BMI.

Furthermore, the inclusion of only clinical cases of diabetes rather than undiagnosed cases of diabetes might have limited our results if the prevalence of undiagnosed cases of diabetes differed substantially among tea categories. We have no indication, however, that this differential misclassification in the outcome was likely.

In conclusion, we observed a linear inverse association between tea consumption and incidence of type 2 diabetes. People who drink at least 4 cups of tea per day may have a 16% lower risk of developing type 2 diabetes than non-tea drinkers. Whether consumption of all types of tea is associated similarly with lower risk of type 2 diabetes and whether this association is causal should be further investigated.
